# Effectiveness of autogenic training on psychological well-being and quality of life in adults living with chronic physical health problems: a protocol for a systematic review of RCT

**DOI:** 10.1186/s13643-020-01336-3

**Published:** 2020-04-07

**Authors:** Maria Pilar Ramirez-Garcia, Jérôme Leclerc-Loiselle, Christine Genest, Renaud Lussier, Golsa Dehghan

**Affiliations:** 1 Faculty of Nursing, Université de Montréal, 2375 Chemin de la Côte-Sainte-Catherine, Montréal, QC H3T 1A8 Canada; 2 Research Centre of the Centre Hospitalier de l’Université de Montréal, Montréal, Canada; 3 Université de Montréal, Montréal, Canada; 4 Applied Clinical Research Unit of the CHU Sainte-Justine, Montréal, Canada

**Keywords:** Autogenic training, Relaxation, Psychological distress, Depression, Anxiety, Quality of life, Chronic conditions

## Abstract

**Background:**

Autogenic training is a relaxation technique that uses systematic exercises to induce a general disconnection of the organism. It is used in conjunction with conventional medical care as part of disease management to relieve symptoms associated with chronic health problems and to improve well-being. The purpose of this systematic review is to evaluate the efficacy of autogenic training on psychological well-being, quality of life, and adverse effects in people living with chronic physical health problems.

**Methods:**

The methodology used follows the recommendations of the Cochrane Handbook for Systematic Reviews of Interventions. Studies, published up to December 31, 2019, will be identified through searches in the following databases: MEDLINE, Web of Science, EMBASE, SCOPUS, PsychINFO, CINAHL, EBM Reviews, Google Scholar, Dissertations & Theses Global, Open Access Theses and Dissertations, OpenGrey, E-Theses Online Service, Grey Literature Report, eScholarship@McGill, Papyrus, and CorpusUL. All studies of randomized controlled trials that assess autogenic training as an intervention to improve psychological well-being and quality of life in adults aged 18 and older living with one or more chronic physical health problem will be considered eligible. The study selection, the data collection, and the evaluation of the risk of bias will be conducted independently and in duplicate by two reviewers. RoB 2 tool will be used to assess the risk of bias. Discrepancies will be resolved through discussion. A tabular and narrative synthesis of data is planned, and a meta-analysis will be done according to the quality of data. The primary outcomes will be general psychological distress, depression, and anxiety, and the secondary outcomes will be quality of life and adverse effects. The present protocol of systematic review is reporting following MECIR standards for the reporting of protocols and the PRISMA-P recommendations.

**Discussion:**

Autogenic training appears to be a promising therapy to improve psychological well-being and quality of life in people living with chronic physical health problems, but no recent reports have synthesized the available evidence in this population. The results of this review will examine and synthesize the evidence on the benefits and harms of autogenic training on psychological well-being and quality of life in people living with chronic physical health problems, thus supporting the development of best practices for complementary approaches.

**Systematic review registration:**

PROSPERO CRD42018105347.

## Background

An increasing number of adults in the world are living with one or more chronic health problems, such as cancer, diabetes, cardiovascular and chronic respiratory diseases, human immunodeficiency virus (HIV), and mental illness [1–4]. Defined as “diseases, conditions and syndromes that are continuing or occurring again and again for a long time […] and are justifiably emotionally and physically taxing for patients and their caregivers,” [5] chronic health problems affect the health and well-being of many adults [1–4]. People living with chronic physical health problems are significantly more likely to experience psychological distress such as depression and anxiety [6–10] and lower quality of life [11, 12].

Studies have shown that people living with chronic health problems are more likely to use relaxation and other mind-body practices compared to adults without health problems [13–17]. Most use these practices in conjunction with conventional medical care as part of disease management to relieve symptoms associated with the chronic health problem, to improve health and wellness, and to play an active role in their health management [16, 18–20].

Autogenic training (AT) is a standardized relaxation technique developed by Schultz around 1930 that uses the mental repetition of six systematic exercises (heaviness, warmth, calm and regular heart function, self-regulation of respiration, warmth in the upper abdomen area, and agreeable cooling of the forehead) to decrease sympathetic tone and induce a general disconnection of the organism [21–23]. AT is simple to learn and easy to practice following brief training. The repeated practice of the exercises increases the person’s capacity to induce ever-deeper relaxation and fosters the accumulation of therapeutic benefits [21].

Although AT can also be used as a tool in professional psychotherapy [21, 24], most of the evidence available comes from the study of AT as a relaxation technique [25]. The mechanism of action of this relaxation technique lies in the relaxation response, as opposed to the stress response, which involves a complex interplay of the endocrine, immune, neurological, and psychological systems [26]. A systematic review with meta-analysis from 35 randomized controlled trials (RCT) and 25 non-randomized controlled trials (NRCT) published between 1932 and 1999 [23] found positive effects of AT on disease-specific symptoms and psychological distress in people living with several chronic health problems like tension headaches/migraine, hypertension, asthma, pain, anxiety, mild-to-moderate depression, and sleep disorders. Even though other RCTs have been conducted since 2000 [27, 28] to assess the effects of AT, no systematic review has been done on the effectiveness of AT on psychological well-being in people living with a chronic physical health problem. Furthermore, the review of Stetter and Kupper [23] did not consider AT to be the only therapeutic component of the intervention. Given that there are more RCTs than NRCTs that assess the effectiveness of AT and that including NCRTs would increase the risk of bias [29], we have decided to include only RCTs for this systematic review. This review will gather additional data that provide further knowledge on the effectiveness of AT on psychological well-being, which includes general psychological distress, depression and anxiety, and quality of life for this target group.

## Objectives

The aim of this systematic review is to evaluate the efficacy of AT on psychological well-being, quality of life, and adverse effects in people living with chronic physical health problems. To this end, the proposed systematic review will answer the following question:

Among people living with chronic physical health problems, what are the effects of AT on general psychological distress, depression, and anxiety (primary) and on quality of life and adverse effects (secondary) compared to control conditions or other treatments?

## Methods

The methodology used for this systematic review follows the recommendations of the Cochrane Handbook for Systematic Reviews of Interventions [30]. We report the protocol of this systematic review based on the Methodological Expectations of Cochrane Intervention Reviews (MECIR) reporting guidance for Cochrane Review protocols [31] and the Preferred Reporting Items of Systematic Reviews and Meta-Analysis for Protocols (PRISMA-P) [32, 33]. The completed PRISMA-P checklist is included as an additional file (see Additional file 1). We registered this protocol in the International Prospective Register of Systematic Reviews (PROSPERO) under the registration number CRD42018105347. If we need to amend this protocol, we will provide the date of each amendment, describe the change, and give the rationale in this section.

## Criteria for considering studies for this review

### Types of studies

We will include parallel, cross-over, and cluster RCTs of AT in people living with chronic health problems. We assume most of the studies will be parallel-group trials. NRCTs will be excluded because most studies evaluating the effectiveness of AT are RCTs. Moreover, according to the *Cochrane Handbook for Systematic Reviews of Interventions*, randomization is the only way to prevent differences between groups at baseline and including NCRTs would increase the risk of bias [29].

### Types of participants

We will include studies that assess AT as an intervention for clinically defined group of adult patients, aged 18 and older living with one or more chronic physical health problems. Since the definition of “chronic disease” and the diseases included within the term vary tremendously, we will use Bernell and Howard proposition [5]. These authors consider chronic health problems as “diseases, conditions and syndromes that are continuing or occurring again and again for a long time, and when taken together affect a large number of individuals who can be quite costly to manage and are justifiably emotionally and physically taxing for patients and their caregivers” [5] (p. 3). A comprehensive list of search terms for chronic conditions will be used as a guide for conducting our review [34–36]. The specific chronic physical health problems that we will include in this review are arthritis, rheumatoid arthritis and osteoarthritis, cancer, cardiovascular and chronic respiratory diseases, cerebrovascular diseases, chronic fatigue syndrome, fibromyalgia, diabetes mellitus, irritable bowel syndrome and colitis, migraine and tension headaches, multiple sclerosis, neurodegenerative diseases like Parkinson and Huntington disease, recurring lower back pain, and viral diseases like HIV/AIDS and hepatitis. We will exclude studies that assess AT in people living with mental disorders. We will also exclude studies involving people living with cancer undergoing chemotherapy, radiotherapy, or surgery, since they might interfere with the outcomes. We will consider studies addressing both adults and children if the data provided for adults are reported separately.

### Types of interventions

#### Experimental

To be included in the review, AT intervention must include the six exercises in the standard combination and sequence (1—heaviness, 2—warmth, 3—calm and regular heart function, 4—self-regulation of respiration, 5—warmth in the upper abdomen area, and 6—agreeable cooling of the forehead) and propose daily practice. AT may be taught in a group or individually. We will exclude studies in which AT is practiced without any training. No limitation according to the length or the place of the intervention will be considered. AT must be the only therapeutic component in one group. Studies where AT is used in combination with another therapy will be excluded. If the intervention is not sufficiently described, we will contact the authors. We will ask them open-ended questions to obtain information about the study and reduce the risk of overly positive answers [37]. The information obtained by the authors will be evaluated independently and in duplicate to assess the eligibility of the study regarding criteria. Discrepancies will be resolved through discussion.

#### Comparator

We will include studies in which the comparator is “no intervention,” “usual care,” or an alternative intervention or treatment. We will exclude studies in which the comparator is an AT intervention with different parameters.

### Types of outcome measures

The primary outcomes include general psychological distress, depression, and anxiety. The secondary outcomes are quality of life and adverse effects. All outcome measure must have been assessed using a validated scale, for example Hospital Anxiety and Depression Scale (HADS), Profile of Mood States (POMS), Hamilton Rating Scales for Anxiety (HAM-a) and Depression (HAM-D), Centre for Epidemiological Studies Depression Scale (CES-D), Spielberger State-Trait Anxiety Inventory (STAI), Ferrans and Powers Quality of Life Index (QLI), and Medical Outcome Study 36-Items Short-Form Health Survey (SF-36). The primary time point for analysis will be the change from the baseline to the end of intervention. We will document any follow-up measurements reported after completion of the intervention in the medium term (up to 3 months after completion of the intervention) or long term (longer than 3 months after completion of the intervention).

### Search methods for identification of studies

#### Electronic searches

In order to identify the primary studies and reviews for answering the research question, we will search for research evidence via different sources: electronic databases, websites, and reference lists. The search strategy for electronic databases was developed from the research question and created and performed by a health sciences librarian with expertise in systematic review searching. The search terms will include all identified variants of “autogenic training” AND all identified variants of “efficacy.” The complete list of search terms is presented in an additional file (see Additional file 2).

We will conduct searches from a total of 16 peer-review and grey literature databases up to December 31, 2019: MEDLINE (all Ovid MEDLINE (R)) 1946 to present, Web of Science [Science Citation Index Expanded (SCI-EXPANDED)—1945 to present/Social Sciences Citation Index (SSCI)—1956 to present/Emerging Sources Citation Index (ESCI)—2015 to present], EMBASE (1974–2019 December 19), SCOPUS, PsychINFO, CINAHL (CINAHL Plus with Full Text), EBM Reviews [Cochrane Central Register of Controlled Trials (CCTR)/Cochrane Database of Systematic Reviews (CDSR)/Database of Abstracts of Reviews of Effects (DARE)/Health Technology Assessment (HTA)], Google Scholar, Dissertations & Theses Global, Open Access Theses and Dissertations (OATD), OpenGrey, E-Theses Online Service (EThOS), The New York Academy of Medicine – The Grey Literature Report, eScholarship@McGill (McGill University), Papyrus, and CorpusUL. The goal will be to conduct a sensitive rather than a specific search of the literature. No limitations will be applied for publication date or language.

We will also search for registered trials at the World Health Organization’s International Clinical Trials Registry Platform Search Portal (www.who.int/trialsearch/) and the US National Institutes of Health’s ongoing trials registry, ClinicalTrials.gov (www.clinicaltrials.gov).

The search results will be entered into the EndNote X8 reference management software for screening, and duplicate records of the same report will be removed. Finally, we will verify the reference lists of all the relevant studies and review the articles to identify additional relevant studies.

### Data collection and analysis

#### Selection of studies

To identify potentially relevant studies, three reviewers (MPRG, JLL & CG) will examine independently and in duplicate titles and abstracts using an eligibility checklist with the specific eligibility criteria from EndNote X8. They will code them as “retrieve” (eligible, potentially eligible or unclear) or “do not retrieve.” Prior to the formal screening process, the same three reviewers will pilot the eligibility criteria on a sample of 10 to 12 papers to clarify the criteria and ensure that they will be applied consistently by the reviewers.

We will retrieve the full-text study reports of the studies coded as “retrieve,” and the same three reviewers (MPRG, JLL, and CG) will screen independently and in duplicate them for inclusion and record the reasons for non-inclusion. Discrepancies will be resolved through discussion. All studies meeting the inclusion criteria will be eligible for the systematic review. A PRISMA flow diagram [32] will be used to document the study selection process.

#### Data extraction and management

We will develop an electronic data extraction form using Microsoft Excel to extract data from the full-text articles. The data form will include study ID (first author, publication year), country, aim, study design, total study duration, sequence generation, allocation sequence concealment, blinding, participants (total number of participants, setting, diagnosis or health problem, age, and sex), total number of intervention groups, AT intervention details (content and length of intervention, number of sessions, provider, and method of delivery), comparator intervention details (content and length of intervention, number of sessions, provider, and method of delivery), adverse effects, outcomes and time points, outcome tools, number of participants in each intervention group, completion of intervention, missing participants, estimate of effect with confidence interval, authors’ conclusions, funding sources and declarations of interest, reference to other included studies, correspondence required, and comments by the reviewer. For each dichotomous outcome, we will extract the numbers from both outcome categories for each intervention group. If this information is not available, we will extract the odds ratio (OR), risk ratio (RR), or risk difference (RD) with a 95% confidence interval or an exact *P* value. For each continuous outcome, we will extract the mean value, the standard deviation, and the number of participants in each intervention group. If this information is not available, we will extract a mean difference or standardized mean difference with a 95% confidence interval or an exact *P* value [38].

We will pilot the data extraction form on a sample of three to six papers to ensure that the form is adequate and that data are extracted consistently. Then, data will be extracted by three reviewers (MPRG, JLL, and CG), independently and in duplicate. Discrepancies will be resolved through discussion. If necessary, reviewers will contact the authors of the selected studies by email (two email attempts) for missing information. Data from multiple reports from a same study will be extracted separately and combined thereafter. One review author (MPRG) will transfer the data into Review Manager (RevMan version 5.3) software. A second review author (JLL) will check the study characteristics entered into Review Manager for accuracy.

#### Assessment of risk of bias in included studies

We will use the version 2 of the Cochrane risk-of-bias tool, RoB 2, for assessing the risk of bias of each outcome for each included studies in five domains: bias from the randomization process, bias due to deviations from intended interventions, bias due to missing outcome data, bias in measurement of the outcome, and bias in selection of the reported result [39]. Based on the responses to signaling questions and algorithms from this tool, we will judge each domain to be “low risk of bias,” “some concerns,” or “high risk of bias.” We will provide written justifications for each judgment in the risk-of-bias table. Finally, we will reach an overall risk of bias judgment for each specific outcome based on judgments for each domain [37, 39].

Three reviewers (MPRG, JLL, and CG) will assess the risk of bias on a pilot sample of three to six papers to ensure that the criteria are applied consistently [37]. Then, the same three reviewers (MPRG, JLL, and CG) will assess the risk of bias of the included studies, independently and in duplicate. Discrepancies will be resolved through discussion. Study authors will be contacted if there is insufficient detail to assess the risk of bias. We will ask them open-ended questions to obtain information about the study and reduce the risk of overly positive answers [37].

### Measures of treatment effect

#### Dichotomous data

For dichotomous data, we will present the results as a summary odds ratio (OR) or a risk ratio (RR) with 95% confidence interval.

#### Continuous data

For continuous data, we will use the mean difference (MD) if outcomes were measured in the same way between trials. We will use the standardized mean difference (SMD) to combine trials that measured the same outcome but used different methods. We will also use the SMD to combine trials that measured the same outcome but used different scales.

#### Unit of analysis issues

In accordance with Higgins et al. [40], for the cross-over trials, we will include only data from the first period and analyze these as if the trial were a parallel-group trial. We will perform separate analysis for outcomes in the short term (immediately after the intervention), medium term (up to 3 months after completion of the intervention), and long term (longer than 3 months after completion of the intervention).

If we have repeated measures of primary outcomes, we will select the immediate after intervention time point measure. In the case of multi-arm studies, in accordance with Higgins et al. [40], we will combine groups to create a single pairwise comparison.

If we identify cluster randomized trials for inclusion, we will include them in the analysis along with individually randomized trials. We will adjust their sample sizes using the methods described in the Cochrane Handbook [40], using an estimate of the intracluster correlation coefficient (ICC) derived from the trial (if possible), from a similar trial, or from a study of a similar population. If we use ICCs from other sources, we will report this and conduct sensitivity analysis to investigate the effect of variation in the ICC. If we identify both cluster randomized trials and individually randomized trials, we plan to synthesize the relevant information. We will consider it reasonable to combine the results from both if there is little heterogeneity between the study designs and the interaction between the effect of intervention and the choice of randomization unit is considered to be unlikely.

We will also acknowledge heterogeneity in the randomization unit and perform a sensitivity analysis to investigate the effects of the randomization unit.

#### Dealing with missing data

We will contact the authors to verify key study characteristics and obtain missing numerical outcome data. If we are not able to obtain the data, we will conduct a sensitivity analysis to evaluate the impact of including such studies in the overall assessment.

#### Assessment of heterogeneity

Heterogeneity was assessed both by visual inspection of the forest plots and a formal statistical test, i.e., *I*^2^ statistic. If high levels of heterogeneity are identified, i.e., *I*^2^ > 50% [41], we will explore the possible between-study sources of heterogeneity using subgroup analysis. More details of subgroup analysis are mentioned in the “Subgroup analysis” section. A random-effects meta-analysis will also be employed as appropriate.

#### Assessment of risk of bias due to missing results

If we have at least 10 studies in the meta-analysis, we will explore possible selective outcome reporting and publication biases using a funnel plot to quantify the potential presence of publication bias [42]. The choice of test for funnel plot asymmetry will depend on the degree of heterogeneity observed.

#### Data synthesis

We will summarize the main characteristics of each included study (population, characteristics of intervention and comparator, outcomes, time points, data, and effect) in a table in order to determine which studies will be grouped for each comparison [43]. For each comparison, we will describe the direction and the size of the effect, the consistency of the effect across studies, and the strength of the evidence for the effect. If tests of heterogeneity are not significant, the fixed-effect model will be perform using Mantel-Heanszel method and weighted MD/or SMD for dichotomous and continuous outcomes, respectively. If statistical heterogeneity is observed (i.e., *I*^2^ > 50%), we will investigate the heterogeneity analysis and consider the usefulness of random-effects model in order to take into account the methodological variation across studies. We will analyze the study trials in two scenarios, and AT will be compared with either a control group/or another treatment or intervention. All *p* values will be two-sided. All statistical analysis will be conducted using Review Manager 5.3 (RevMan 2014) with the DerSimonian and Laird random-effects model [41].

#### Quality of evidence

Three reviewers (MPRG, JLL, and CG) will assess the quality of evidence for each outcome using the GRADE approach, independently and in duplicate [44]. This will involve consideration of within- and across-study risk of bias, consistency of effect, directness of evidence, precision of the effect estimates, and publication bias. The reviewers will grade the quality of evidence for each outcome as “high,” “moderate,” “low,” or “very low” and justify and document their assessment. Discrepancies will be resolved through discussion.

#### Subgroup analysis

If appropriate data are available, we will further investigate sources of heterogeneity through the following subgroup analysis:

1. By chronic health problem (see the “Types of participants” section)
2. By length of intervention (at least 8 weeks; 8 weeks or more)
3. By type of training (group or individual)

#### Sensitivity analysis

If appropriate, we will conduct sensitivity analysis to investigate the robustness of our findings. We will repeat the analysis after excluding studies with missing numerical outcome data. We will also repeat the analysis after excluding cross-over trials and trials with a high risk of bias.

## Discussion

AT could be a promising therapy to improve psychological well-being and quality of life among people living with chronic physical health problems. In a context where there are more and more people living with one or more chronic health problems, the use of a relaxation technique like AT could contribute to their whole health process [45]. However, no recent reports have summarized the available evidence in this population.

This systematic review will examine and synthesize the evidence for the benefits and harms of AT on psychological well-being and quality of life in people living with physical chronic health problems. In accordance with Schünemann and his colleagues and on behalf of the GRADEing Methods Group [46], the conclusions will present implications for practice and for research. For practice, we will describe the quality of the evidence, the balance of the benefits and harms, and other factors that might influence a decision in this area such as values and preferences, costs, and availability of resources. We will use the EPICOT format (evidence, population, intervention, comparison, outcome, and time stamp) and the GRADE guidelines to report the research implications. The findings of this systematic review will be published in a peer-reviewed journal and disseminated to the public.

## Supplementary information

**Additional file 1.** PRISMA-P 2015 checklist.

**Additional file 2.** Search strategy and terms used for Medline database.

## Data Availability

The dataset generated and analyzed during the current study will be available from the corresponding author on reasonable request.

## Abbreviations

AT: Autogenic training
EPICOT: Evidence, Population, Intervention, Comparison, Outcome and Time stamp
GRADE: Grading of Recommendations Assessment, Development and Evaluation
HIV: Human immunodeficiency virus
ICC: Intracluster correlation coefficient
MD: Mean difference
MECIR: Methodological Expectations of Cochrane Intervention Reviews
NRCT: Non-randomized controlled trial
OR: Odds ratio
PRISMA-P: Preferred Reporting Items for Systematic Review and Meta-Analysis Protocols
PROSPERO: Prospective Register of Systematic Reviews
RCT: Randomized controlled trial
RD: Risk difference
RR: Risk ratio
SMD: Standardized mean difference
