# Supplementary material for: Effectiveness of autogenic training on psychological well-being and quality of life in adults living with chronic physical health problems: a protocol for a systematic review of RCT
Source: Syst Rev. 2020 Apr 7;9:74. doi: 10.1186/s13643-020-01336-3 (PMC7137438; doi:10.1186/s13643-020-01336-3)
Supplement: Supplementary file 2 — Additional file 2. Search strategy and terms used for Medline database. [file 13643_2020_1336_MOESM2_ESM.docx]

**Additional file 2:** Search strategy and terms used for Medline database.

MEDLINE (All Ovid MEDLINE(R) 1946 to Present)

1. ("autogenic training" OR "autogenic trainings" OR "autogene training" OR "autogene trainings" OR "autogenous training" OR "autogenous trainings" OR "autogenic meditation" OR "autogene meditation" OR "autogenous meditation" OR "autogenic relaxation" OR "autogene relaxation" OR "autogenous relaxation" OR "autogenic therapy" OR "autogene therapy" OR "autogenous therapy").ti,ab
2. ("autogenic training").sh
3. 1 OR 2
4. (application* OR benefit* OR change* OR compare* OR comparison* OR consequence* OR decrease* OR effect* OR efficaciousness OR efficacy OR efficien* OR enhance* OR evaluat* OR impact* OR improv* OR increas* OR influence* OR optimiz* OR outcome* OR reduc* OR result* OR treatment*).ti,ab
5. ("Evaluation Studies as Topic" OR "Evaluation Studies [Publication Type]").sh
6. 4 OR 5
7. 3 AND 6
8. 2000:2018.(sa_year)
9. 7 AND 8
